# Covariance statistics and network analysis of brain PET imaging studies

**DOI:** 10.1038/s41598-019-39005-8

**Published:** 2019-02-21

**Authors:** Mattia Veronese, Lucia Moro, Marco Arcolin, Ottavia Dipasquale, Gaia Rizzo, Paul Expert, Wasim Khan, Patrick M. Fisher, Claus Svarer, Alessandra Bertoldo, Oliver Howes, Federico E. Turkheimer

**Affiliations:** 1 Department of Neuroimaging, IoPPN, King’s College London, London, United Kingdom; 2 Department of Information Engineering, University of Padova, Padova, Italy; 3 Invicro UK, London, United Kingdom; 4 Department of Mathematics, Imperial College London, London, United Kingdom; 5 EPSRC Centre for Mathematics of Precision Healthcare, Imperial College London, London, United Kingdom; 6 Florey Institute of Neuroscience and Mental Health, Melbourne Brain Centre, Melbourne, Australia; 7 Neurobiology Research Unit, Copenhagen University Hospital Rigshospitalet, Copenhagen, Denmark; 8 Department of Psychosis studies, IoPPN, King’s College London, London, United Kingdom

## Abstract

The analysis of structural and functional neuroimaging data using graph theory has increasingly become a popular approach for visualising and understanding anatomical and functional relationships between different cerebral areas. In this work we applied a network-based approach for brain PET studies using population-based covariance matrices, with the aim to explore topological tracer kinetic differences in cross-sectional investigations. Simulations, test-retest studies and applications to cross-sectional datasets from three different tracers ([^18^F]FDG, [^18^F]FDOPA and [^11^C]SB217045) and more than 400 PET scans were investigated to assess the applicability of the methodology in healthy controls and patients. A validation of statistics, including the assessment of false positive differences in parametric versus permutation testing, was also performed. Results showed good reproducibility and general applicability of the method within the range of experimental settings typical of PET neuroimaging studies, with permutation being the method of choice for the statistical analysis. The use of graph theory for the quantification of [^18^F]FDG brain PET covariance, including the definition of an entropy metric, proved to be particularly relevant for Alzheimer’s disease, showing an association with the progression of the pathology. This study shows that covariance statistics can be applied to PET neuroimaging data to investigate the topological characteristics of the tracer kinetics and its related targets, although sensitivity to experimental variables, group inhomogeneities and image resolution need to be considered when the method is applied to cross-sectional studies.

## Introduction

The human brain is an extraordinary system with a distinct anatomical architecture and a complex functional organisation^1,2^. Since the nineteenth century it became clear that neuronal activity is organised into well-defined networks allowing both segregation and integration of information^3^. Such networks are thought to provide the physiological basis of brain cognitive functions and mental representations^4–6^.

Following the development of complex system science, graph theory has become an important tool for the analysis of structural and functional neuroimaging data acquired with different modalities including MRI, EEG and MEG^7^. In fact, it has significantly contributed to improve our understanding of the topological properties of brain functional and structural organization in both normal and pathological conditions^8,9^.

A graph is a mathematical object able to represent spatially distributed but topologically linked elements. The basic structure to calculate the higher order of its associated network parameters is the adjacency matrix, which is estimated by compiling all pairwise associations between different brain regions of interest (ROIs)^7^. Depending on the data under exam, the adjacency matrix provides different information about the link between ROIs: for example, in the fMRI analysis it expresses the temporal correlations of the fMRI signal between ROIs, while when using DTI data it expresses the number of tracts or streamlines connecting the ROIs^7^. A common characteristic of these functional and structural analyses is the possibility to obtain an adjacency matrix per subject, which consequently allows to estimate higher-order metrics at the individual level and perform statistical comparisons between groups.

As regards molecular imaging modalities such as PET, the use of graph theoretical approaches has been hindered by the static nature of the acquired data. In fact, a PET imaging session returns only a single representation of the investigated biological processes irrespective of the duration of the imaging session. As a result, it becomes quite challenging to estimate subject-specific adjacency matrices and consequently derive individual graph metrics. A way to overcome the lack of repeated PET measures at the subject level has been proposed by Horwitz and colleagues in 1984^10^ to study the human brain metabolic connectivity at rest and in pathological conditions^11–14^. The authors implemented a between-subject interregional correlation analysis (IRCA), under the assumptions that brain regions with significantly correlated [^18^F]FDG uptake are functionally interconnected, being the strengths of these connections proportional to the magnitude of their correlation coefficients, and that this pattern is representative of the entire population of subjects used for the analysis^10^. This approach can be compared to the seed-based analysis used in fMRI as it consists in extracting the mean [^18^F]FDG values from a seed of interest for all subjects and using these values as a covariate in a general linear model to find the regions showing significant correlations across subjects. This method has been applied at the regional and voxel levels^15–17^ to investigate metabolic connectivity^16,18–21^ and extended to other PET targets, such as neuroreceptor systems or enzymatic rates^22–25^. These studies have confirmed the consistency of PET measures at the population level and suggested that extending the graph-based approaches to PET studies would lead to a significantly deeper understanding of brain’s architectural complexity as well as group-specific biological alterations occurring in pathological conditions.

In this context, our study aims to assess the general applicability of graph-based analysis to brain PET data and to validate the statistical methods to be used in cross-sectional studies (e.g. different groups or longitudinal imaging sessions). To achieve these purposes, we used covariance statistics and network-derived metrics to investigate the topological characteristics of the biological functions measured by different PET tracers ([^18^F]FDG for glucose metabolism, [^18^F]FDOPA for dopamine synthesis and [^11^C]SB217045 for serotonin 5HT4 receptor density) and compared them across groups in a similar way as done in anatomical covariance analysis^26^. Data from more than 400 subjects were used. In the first part of this study, we focused on the methodological validation of the approach by using data resampling to assess false positive rates and test-retest data analysis to assess its reproducibility. We also measured the method sensitivity to some experimental variables, i.e. the impact of scanner type and PET data partial volume on the covariance analysis. In the second part, we tested its clinical applicability on Alzheimer’s Disease (AD) PET data. Previous evidences have consistently shown that AD pathology is associated with the presence of both hypometabolism and alteration of brain metabolic connectivity^27–30^. Therefore, by running a cross-sectional analysis on the Alzheimer’s Disease Neuroimaging Initiative (ADNI) PET dataset^31^, we evaluated the ability of the graph-based method to complement the results obtained with the traditional approaches.

## Theory

### Definition of PET adjacency matrix and high-order metrics

**Figure 1:**
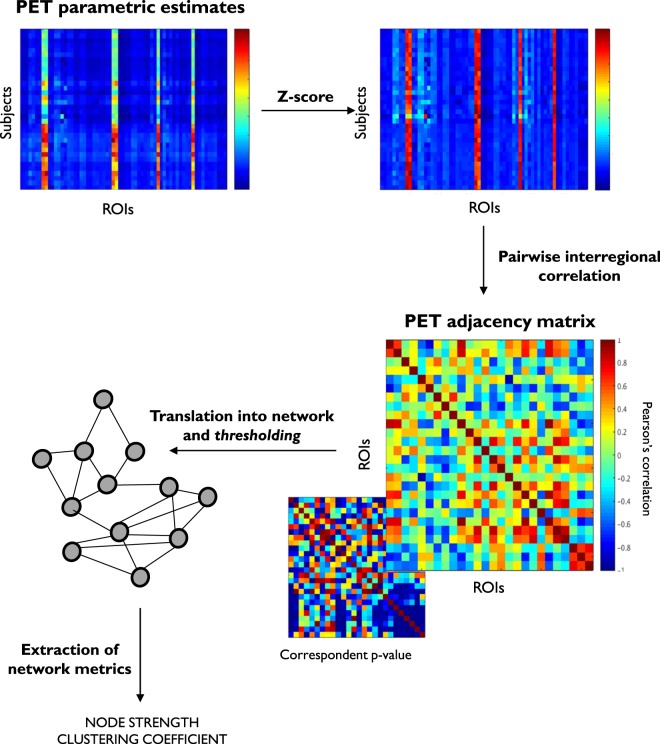
Population PET adjacency pipeline. Representative regional subject estimates are transformed into z-score to remove inter-subject differences of the tracer uptake in mean and standard deviation. For each couple of regions interregional correlations are computed across subjects together with the correspondent p-values and used to define the PET adjacency matrix. This is translated into a network, where nodes are the ROIs and interregional correlations the links. Thresholding is applied to preserve the strongest functional connections^37^ before any network metric is extracted as representation of the biological organisation of the PET tracer across the brain.

The population-specific PET adjacency matrices were estimated by extracting for each scan the mean tracer kinetic estimates within each ROI and calculating the pair-wise linear correlation across subjects (Fig. 1). Importantly, before the population covariance matrix is computed, individual kinetic estimates undergo a z-score transformation to remove inter-subject differences of the tracer uptake in mean and standard deviation^10^. The resulting mathematical object is a *N* × *N* symmetric matrix (where *N* is the number of ROIs used for the tracer quantification) which provides information about the spatial organization of the biological function measured by the injected radiotracer. To gain a clear biological meaning, the method requires the use of a *homogenous* group of PET scans, similar in terms of experimental design, quantification pipeline and subject characteristics. Any variation in one of these variables is likely to impact the PET adjacency matrix in an unpredictable way.

Graph-based approaches can then be applied to highlight the core characteristics of these weighted matrices by returning a set of meaningful computable measures (also called *network metrics*), which allows to assess the between-region interactions as well as the identification of functionally connected nodes. Since the main interest of this work is to characterise the overall organisation of the biological function expressed by the PET adjacency matrix, we decided to focus our analysis on the node strength and the clustering coefficient. These two metrics have already been used in PET neuroimaging research^32^ and guarantee a certain level of result interpretability, being consistent with the static nature of PET measures. In fact, in absence of a dynamic exchange of information between the nodes of a PET network, the application of dynamic metrics such as the betweenness centrality would be of difficult interpretation. Both node strength and clustering coefficient were defined according to the Brain Connectivity Toolbox (BCT)^33^:

- *Node strength* is the average connectivity of a node and is defined as the sum of all neighbouring link weights.
- *Clustering coefficient* is a measure of functional segregation and quantifies the number of connections that exist between the nearest neighbours of a node as a proportion of the maximum number of possible connections^34^. It accounts for the number of triangles in the network, with a high number of triangles implying segregation.

Graph metrics were extracted from the PET weighted covariance matrices after thresholding. Thresholding is commonly used in MRI analysis^35,36^ to preserve the strongest functional connections^37^. For comparative reasons, the distribution of the interregional correlations identified by the inferior triangle of the PET adjacency matrix was also measured, excluding the elements of the principal diagonal. No thresholding was applied in this analysis to avoid threshold-driven alternations in the shape of interregional correlation distribution and its number of elements. All the network metrics and interregional correlations were analysed after Fisher’s r-to-z transform of the PET adjacency matrix, as commonly performed in the literature^38^.

### Statistical tests for the between-group comparison of adjacency matrices and higher-order metrics

Measuring the topological characteristics of a PET adjacency matrix would not be informative without the possibility to compare this mathematical object between groups. Hence, several statistical tests were implemented to compare the PET adjacency matrices and the graph metrics.

The node strengths and the node clustering coefficients, as well as the distribution of PET correlation values, were compared in terms of mean and variance using the Welch’s and F statistics respectively. These tests were implemented assuming a normal data distribution (*parametric test*) or with 10,000 permutations (*permutation test*) to generate the null distribution from the data without any a priori hypothesis^39,40^.

In addition to these analyses, we also included the Krzanowski’s test on the principal components of the PET adjacency matrices to investigate the equality of their eigenvectors and eigenvalues^41^ and a test on functional entropy^42^ of the PET adjacency matrix as a global measure for the uncertainty of the system. Full details on the methods for both Krzanowski’s and entropy test are reported in the supplementary material.

## Methods

### Method validation

Three different approaches were used to perform the methodological validation of the statistical tests used to compare the PET adjacency matrices and their associated graph-metrics: (1) data resampling (without repetition), (2) test-retest data analysis and (3) sensitivity analysis to experimental variables.

#### Resampling test

This test aimed at generating groups with similar properties to investigate the reliability of parametric vs permutation tests and the impact of thresholding the PET adjacency matrix on cross-sectional comparisons. The main idea of the test is that two random groups generated from the same homogenous population should not be different between each other.

Three different PET datasets corresponding to different radiotracers (i.e. [^18^F]FDG, [^18^F]FDOPA and [^11^C]SB207145) acquired in a homogenous group of healthy controls were used for this analysis. A summary of the dataset sizes and tracer biological targets is reported in Table 1. Please note that the use of different PET tracers allowed to test different distributions of PET tracer uptakes across the brain, different segmentation methods and different kinetic parameters. The ROIs were selected consistently with the segmentation approaches previously employed for these radiotracers because they have been shown to be relevant for the tracer uptakes (Table 1). The regional kinetic estimates of each dataset were randomly halved into two groups of equal number of scans and the corresponding PET covariance matrices as well as their associated PET metrics were statistically compared. The randomisation process was defined from all the possible data combinations (with no repetition) but limited to 10,000 cases. For all these tests no difference between the two groups was assumed as the groups were derived from the same homogenous population. The false positive rate (FPR) was hence used as performance index. In accord with the chosen statistical threshold (5%), we expected a similar FPR as target.

**Table 1:** Datasets for method validation.

Tracer	[^18^F]FDG	[^18^F]FDOPA	[^11^C]SB207145
*Biological target function*	Cerebral metabolic rate for glucose	Dopamine Synthesis capacity	serotonin 5TH4 receptor availability
*Parameter of interest*	CMRgl	${K}_{i}^{cer}$	*BP*_*nd*_
*Number of scans*	80	52	60
*Number of ROIs*	23	46	67
*Dataset Reference*	PET NMRC summaries - Banner Alzheimer’s Institute (Arizona) from Alzheimer’s Disease Neuroimaging Initiative (ADNI)^31^	This population of healthy controls was derived from an in-house database. Full details on experimental design and data analysis are reported in^74^	This population of healthy controls was derived from a CIMBI database^46^. Full details on experimental design and data analysis are reported in^47,75^

The graph metrics (node strength and clustering coefficient) were estimated after thresholding the PET covariance matrices with different thresholds (range [0.1:0.6]) to test the dependency of these parameters on thresholding. This range of thresholds has been used only for the resampling test as a proof of concept. Also, the matrices were thresholded but not binarized. Statistical testing on functional entropy and principal components was applied without thresholding the PET covariance matrices.

The same resampling procedure was used to assess the method sensitivity to the number of subjects used to define the PET covariance matrix. For this analysis the population size was reduced to 10 subjects per group, which is a sample size found in many cross-sectional PET studies of the past years^43^.

#### Test-retest analysis

Two different datasets were used for this validation. The first dataset was made of 8 healthy subjects (5 males and 3 females) who received two [^18^F]FDOPA PET scans two years apart^44^. The tracer was administered intravenously by bolus injection (approximately 150MBq) and activity measured dynamically for 95 minutes. The main parameter of interest was ${K}_{i}^{cer}$ as a proxy of the dopamine synthesis capacity obtained using cerebellum as reference region^45^. The second dataset consisted of 35 subjects (24 males and 11 females) scanned twice with [^11^C]SB207145 and was obtained from the publicly available repository at the Centre for Integrated Molecular Brain Imaging (CIMBI)^46^. In this dataset, the main parameter of interest was the tracer binding potential (*BP*_*nd*_) as a proxy of the serotonin 5TH_4_ receptor availability^47^. Details on the tracer synthesis, PET experimental design, subject demographic and quantification pipeline are described extensively elsewhere (see reference^44^ for [^18^F]FDOPA and^46^ for [^11^C]SB207145 tracer). For both datasets, no change in any of the experimental variables was applied between test and retest conditions, and a good reproducibility of the main parameters of interest (both ${K}_{i}^{cer}$ and *BP*_*nd*_) has been described by previous studies for brain tissues with significant expression of the tracer targets (intra-class correlation coefficient range: 0.68–0.94 for [^18^F]FDOPA; 0.76–0.88 for [^11^C]SB207145)^44,47^. We extracted the mean dopamine synthesis values within 46 ROIs defined by the Hammersmith atlas^48^ and the mean 5HT4 receptor density within the 67 ROIs defined on each subject’s MR images in a user-independent fashion with the PVElab^49^. Then, the adjacency matrices were estimated for each tracer and group by calculating the ROI-to-ROI correlation across subjects. From this step, we also obtained p-value matrices that we used to threshold the adjacency matrices before estimating the graph metrics: all the correlations with a p-value > 0.05 were discarded from the correlation matrix. We also used the absolute value of the matrix to preserve both positive and negative connections between the nodes. Finally, we run the entropy and Krzanowski’s tests on the unthresholded matrices and the Welch’s and F tests on the graph metrics to assess the reproducibility of PET covariance statistics and network metrics between test and retest conditions. Of note, the use of [^18^F]FDOPA and [^11^C]SB207145 allowed a more comprehensive investigation of the PET covariance analysis reproducibility as the two tracers refer to different biological systems (dopamine and serotonin respectively), tissue uptakes (irreversible and reversible respectively), and image analysis pipeline including a different brain tissue segmentation.

#### Sensitivity to experimental variables

The graph method applied to PET data was also validated in terms of its sensitivity to some experimental variables, namely the impact of the scanner type and PET data partial volume on the covariance analysis. Two different datasets from the CIMBI data repository were used. The first compared the [^11^C]SB207145 brain PET scans of 45 subjects acquired with a high-resolution research tomograph (HRRT, FWHM ~ 2-3 mm)^50^ with the [^11^C]SB207145 brain PET scans of 37 subjects acquired with a GE-Advance PET scanner (FWHM ~ 5-6 mm)^51^. The participants (29 male/16 female for HRRT group; 19 male/18 female for GE-Advance group) were recruited under similar inclusion/exclusion criteria. The second dataset consisted of 59 [^11^C]SB207145 brain PET scans (27 male/32 female; 22 HRRT/37 GE Advance). Covariance analysis was performed on the same PET scans with and without partial-volume (PV) correction obtained by using the Muller-Gartner method, with a point spread function of 6 mm for GE Advance data and 3 mm for HRRT data^52^.

In both cases, only healthy controls were included and the PET covariance matrices were created from the regional *BP*_*nd*_ estimates (67 ROIs per subject as for the [^11^C]SB207145 test-retest analysis). Finally, the graph metrics were estimated from the thresholded matrices and cross-sectional statistical comparisons were performed to test whether a higher partial volume as well as a lower PET scanner resolution have an effect on the PET covariance matrix (i.e., higher interregional correlation values), as already reported in previous studies^10^.

### Application to clinical PET data

A cross-sectional analysis on ADNI PET data^31^ was also performed to evaluate the ability of the graph-based method to complement the previous AD findings obtained with the traditional methods. We considered a dataset of 293 [^18^F]FDG PET scans including 76 AD patients, 137 subjects with mild cognitive impairment (MCI) and 80 age-matched controls. The dataset was downloaded from the Banner Alzheimer’s Institute (Arizona) PET NMRC summaries already post-processed as part of the ADNI database (adni.loni.usc.edu). Full details on the experimental protocol and image analysis (including pre- and post-processing) are reported on the ADNI PET website (http://adni.loni.usc.edu/data-samples/pet/), while subject demographics are shown in Table 2.

**Table 2:** [^18^F]FDG Brain PET dataset.

	Healthy Controls	MCI	AD	STATS
*Number of participants*	80	137	76	
*Gender Ratio*	50M/30F	99M/38F	46M/36F	
*Age (years)*	76 ± 5	76 ± 7	76 ± 7	F (2,290) = 0.0305 p = 0.737
*MMSE*	29 ± 1	27 ± 2	23 ± 2	F (2,290) = 222 p < 0.001
*Baseline glucose (mg/dl)*	97 ± 20	100 ± 18	94 ± 22	F (2,290) = 2.4 p = 0.096
*Injected dose (mCi)*	5.5 ± 1.5	5.3 ± 0.9	5.1 ± 0.3	F (2,290) = 2.6 p = 0.073

MCI: mild cognitive impairment; AD: Alzheimer’s Diseases; MMSE: mini-mental state examination.

For each subject, the published rates of glucose metabolism for 23 ROIs were considered. ROIs included hippocampus as well as parietal, temporal, frontal, and posterior cingulate cortices^53^. These estimates were hence used to define the PET adjacency matrices and related graph metrics. Finally, between-group differences were estimated using the statistical tests described in the Theory section which include the Krzanowoski’s test, the mean and the standard deviation to compare the three groups’ PET correlation values distribution, the Welch’s and F statistics for the node strength and clustering coefficient respectively, and the entropy statistic for the different groups’ entropy.

## Results

### Method validation – Resampling test

#### Parametric vs permutation tests

We found a remarkable difference between the permutation and the parametric tests (Table 3): while the permutation tests aligned around the 5% FPR target (mean ± SD: 5.0 ± 0.6%; min: 3.4%; max: 6.0%) irrespective of the metrics used, the parametric tests returned a significantly higher fraction of false positives (mean ± SD: 41.6 ± 18.0%; min: 15.0%; max: 70.4%). The test performances were consistent across tracers. In terms of PET covariance matrix parameters (i.e. correlation distribution, entropy and principal components) and network-derived metrics (i.e. node strengths or clustering coefficients), no differences were found between the two groups.

**Table 3:** Resampling test results.

*PARAMETRIC TESTS*	Correlation	Strength	Clustering
**[** ^**18**^ **F]FDG**			
*PARAMETRIC TESTS*	Correlation	Strength	Clustering
FPR on mean (%)	31.3	21.8	35.2
FPR on variance (%)	24.2	17.9	15.0
*PERMUTATION TESTS*	Correlation	Strength	Clustering
FPR on mean (%)	4.5	5.3	4.6
FPR on variance (%)	5.3	5.0	4.9
*OTHER TESTS*	Entropy	K.’s eigenvectors	K.’s eigenvalues
FPR (%)	4.4	5.7	4.9
**[** ^**18**^ **F]FDOPA**			
*PARAMETRIC TESTS*	Correlation	Strength	Clustering
FPR on mean (%)	54.7	40.1	52.8
FPR on variance (%)	67.2	58.5	42.6
*PERMUTATION TESTS*	Correlation	Strength	Clustering
FPR on mean (%)	4.5	5.7	4.7
FPR on variance (%)	6.0	6.0	5.3
*OTHER TESTS*	Entropy	K.’s eigenvectors	K.’s eigenvalues
FPR (%)	5.2	4.7	3.4
**[** ^**11**^ **C]SB207145**			
*PARAMETRIC TESTS*	Correlation	Strength	Clustering
FPR on mean (%)	68.1	27.9	39.5
FPR on variance (%)	70.4	60.7	21.1
*PERMUTATION TESTS*	Correlation	Strength	Clustering
FPR on mean (%)	4.4	5.9	5.3
FPR on variance (%)	5.5	4.2	4.8
*OTHER TESTS*	Entropy	K.’s eigenvectors	K.’s eigenvalues
FPR (%)	5.0	5.0	4.4

FPR: false positive rate. K.: Krzanowski.

#### Threshold sensitivity analysis

**Figure 2:**
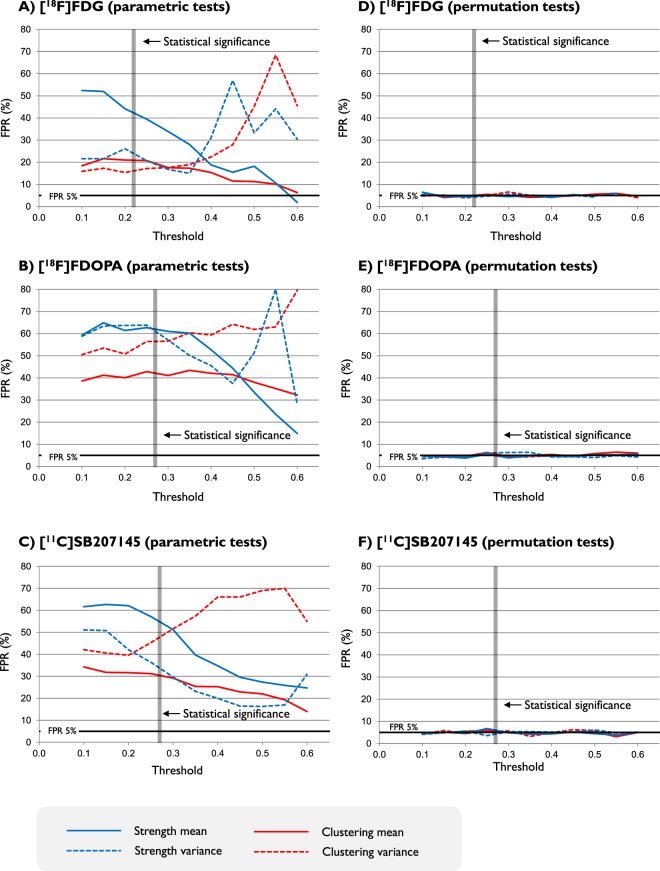
Sensitivity of network metrics to thresholding in parametric and permutation tests. False positive rates (FPRs) are reported as function of different thresholding. The minimum value of correlation in the PET covariance matrix above statistical significant threshold (p-value < 0.05) is also explicitly indicated. As shown by the figure, this value is not constant as it depends on the number of data points used to compute the interregional correlations, which corresponds to the number of subjects used to define the PET adjacency matrix (40 for [^18^F]FDG, 25 for [^18^F]FDOPA and 30 for [^11^C]SB207145). Blue lines refer to node strength, red lines refer to clustering coefficient. Mean and variance analysis are reported in solid and dashed lines respectively. Number of ROIs is 23 for [^18^F]FDG, 45 for [^18^F]FDOPA and 67 for [^11^C]SB207145.

In line with the other results, the FPR from the permutation tests remained stable around 5% for both network metrics in terms of mean and variance, irrespective of the type of tracer (Fig. 2). In contrast, there was a clear effect of the threshold for the parametric tests: in all the cases, no test aligned with 5% FPR target (Fig. 2).

#### Population size sensitivity analysis

**Figure 3:**
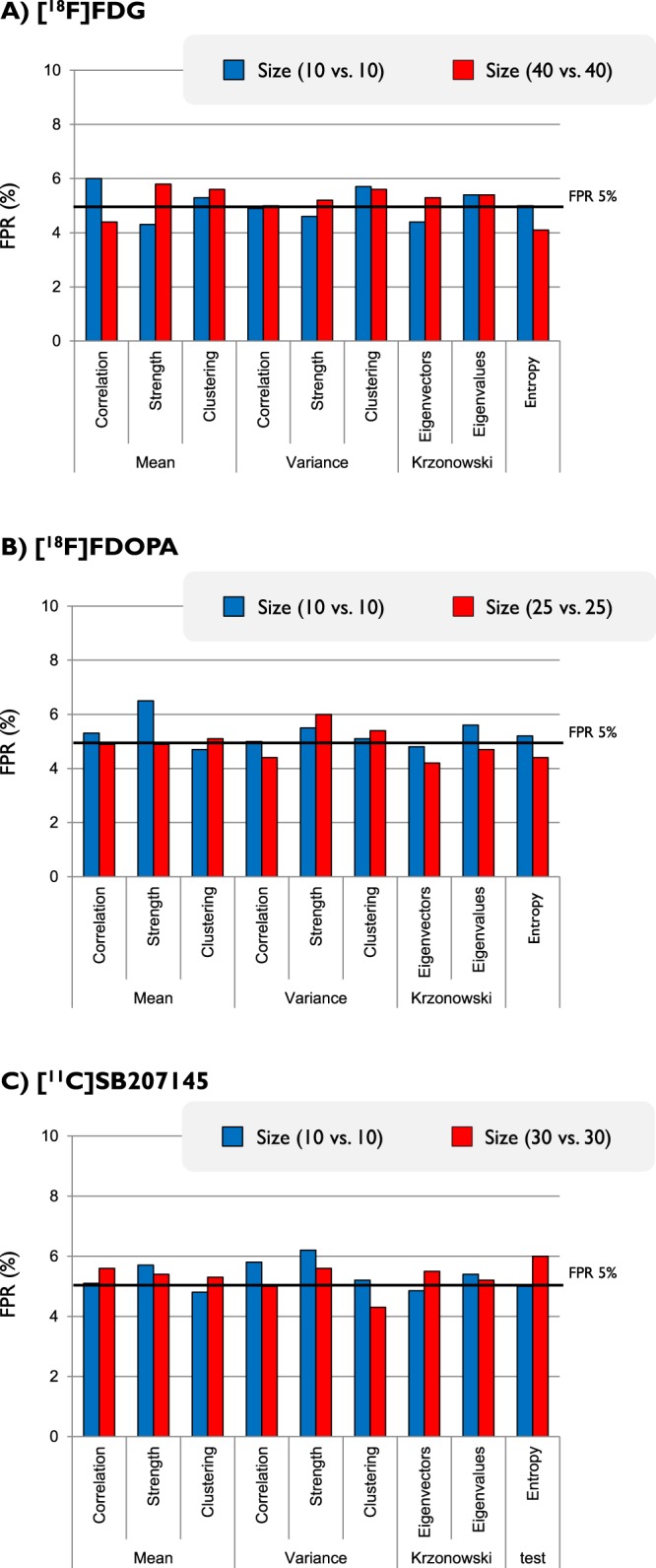
Test sensitivity to population size. False positive rates (FPRs) as obtained for different metrics and different tracers are reported as function of the full dataset (red bars) and the reduced one (10 subjects per groups, blue bars). All the results refer to permutation analysis. Paired t-test between full dataset FPRs and reduced dataset FPRs does not show any statistical difference in any of the tracers (p-value: 0.78 for [^18^F]FDG, 0.15 for [^18^F]FDOPA and 0.94 for [^11^C]SB207145).

PET covariance analysis to test population size is reported in Fig. 3. Parametric tests were excluded for these and all the other analyses as they showed a very high fraction of false positives (FPR > 50%). When the population used to compute the PET adjacency matrix was reduced to 10 subjects, no differences on the test performance were found in comparison to the full dataset size (40 PET scans for [^18^F]FDG, 25 PET scans for [^18^F]FDOPA and 30 PET scans for [^11^C]SB207145). All the tests and metrics were randomly aligned around the 5% FPR target (variability <1%), irrespective of the type of tracer.

### Method validation - Test-retest analysis

**Figure 4:**
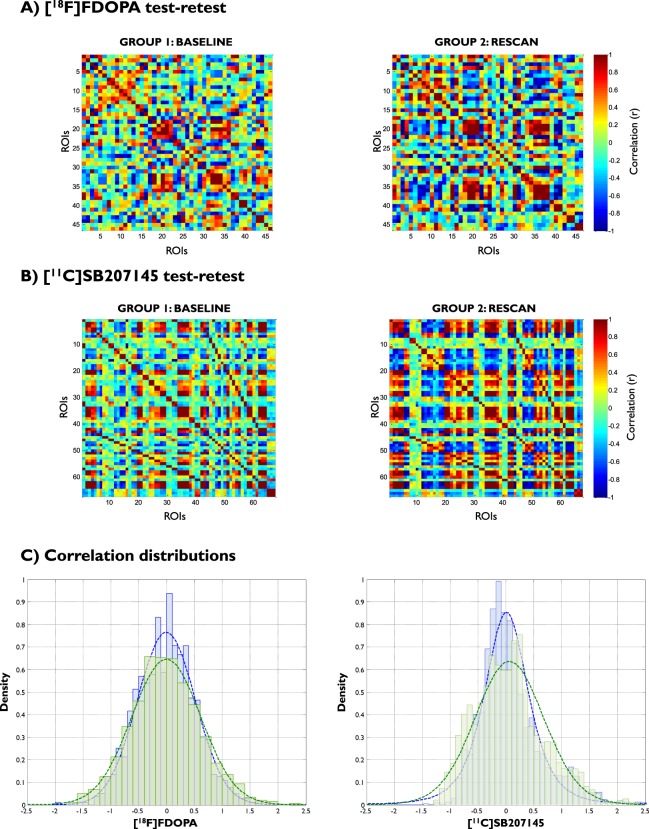
Method reliability analysis. Test-retest analysis of PET adjacency matrices for [^18^F]FDOPA (**A**) and [^11^C]SB207145 (**B**). The distributions of interregional correlations are also reported (**C**). Blue lines and bars refer to baseline group. Green lines and bars refer to rescan group.

For the [^18^F]FDOPA test-retest case (Fig. 4A), intraclass correlation coefficients (ICC) were 0.64 for the correlation distribution, −0.2 for the node strength and −0.27 for the clustering coefficient. When the analysis was limited to the brain regions with non-negligible dopamine synthesis capacity (i.e. ${K}_{i}^{cer}$ > 0.005 min^−1^), ICCs increased to 0.74 for the correlation distribution, 0.52 for the node strength and 0.62 for the clustering coefficient. All the tests on node strength, clustering coefficient and correlation distribution were not significant for both mean and variance. The two groups were similar in terms of entropy (mean relative difference: 8%; p-value = 0.13), eigenvectors (Krzanowski’s *λ*: 10.67) and eigenvalues (Krzanowski’s *μ*: 8.83).

The same results were obtained for the [^11^C]SB207145 test-retest case (Fig. 4B): ICC estimates were 0.93 for correlation distribution, 0.82 for node strength and 0.52 for clustering coefficient. No difference was found for all the tests on node strength, clustering coefficient and correlation distribution for both mean and variance (p-value > 0.05, Fig. 4C). Entropy and principal component tests were also similar between the two groups (entropy mean relative difference: +10%; Krzanowski’s *λ*: 26.39; Krzanowski’s *μ*: 14.22). Full results on PET adjacency matrices and statistical tests are reported in the supplementary methods section under ‘Test-retest analysis’.

### Method validation: Sensitivity to scanner type and image processing

PET covariance analysis was found to be sensitive to the scanner used for the acquisitions. When [^11^C]SB207145 PET data acquired with HRRT were compared with a matched group of subjects acquired with the GE-Advance scanner, significant increases were found for the mean correlation, node strength and clustering coefficient (p-value < 0.01). These parameters were not different in variance (p > 0.95). Entropy was also significantly different (HRRT vs GE-Advance mean relative difference = 30%; p < 0.01). The two PET covariance matrices were also different in terms of principal component eigenvectors (p-value < 0.01; Krzanowski’s *λ*: 24.76; Krzanowski’s *μ*: 24.38)

Similarly, the partial volume correction method used was found to significantly reduce the mean node strength and mean clustering coefficient. Entropy and principal component tests were also sensitive to the use of the partial volume correction (p-value ≤ 0.01). Overall, the quality and resolution of the PET images returned by the scanners consistently affected the characteristics of the PET covariance matrices (Supplementary material - Sensitivity to scanner type and partial volume).

### Application to clinical PET data

**Figure 5:**
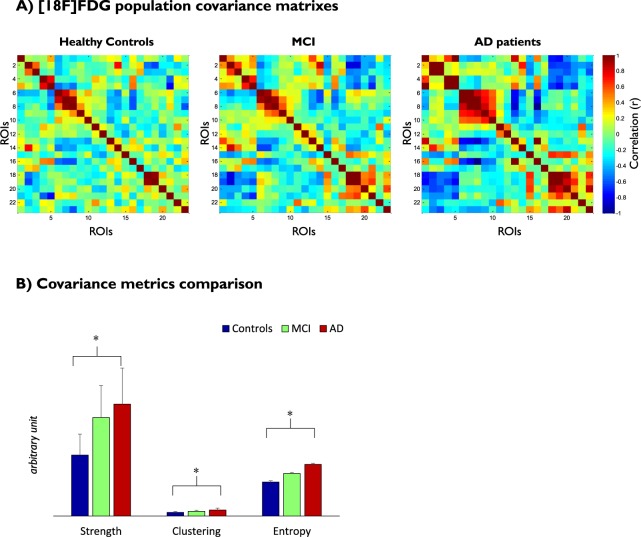
PET covariance analysis in AD. (**A**) PET adjacency matrices in healthy controls and subjects with MCI and AD. ROIs include both left and right hemispheres of hippocampus, superior temporal gyrus, mid-temporal gyrus, inferior temporal gyrus, supramarginal gyrus, fusiform gyrus, parahippocampal gyrus, angular gyrus, inferior parietal gyrus, precuneus, mid-occipital gyrus and cingulate cortex. (**B**) Comparison of network metrics across groups visualised in term of mean and standard deviation (error bars).

**Figure 6:**
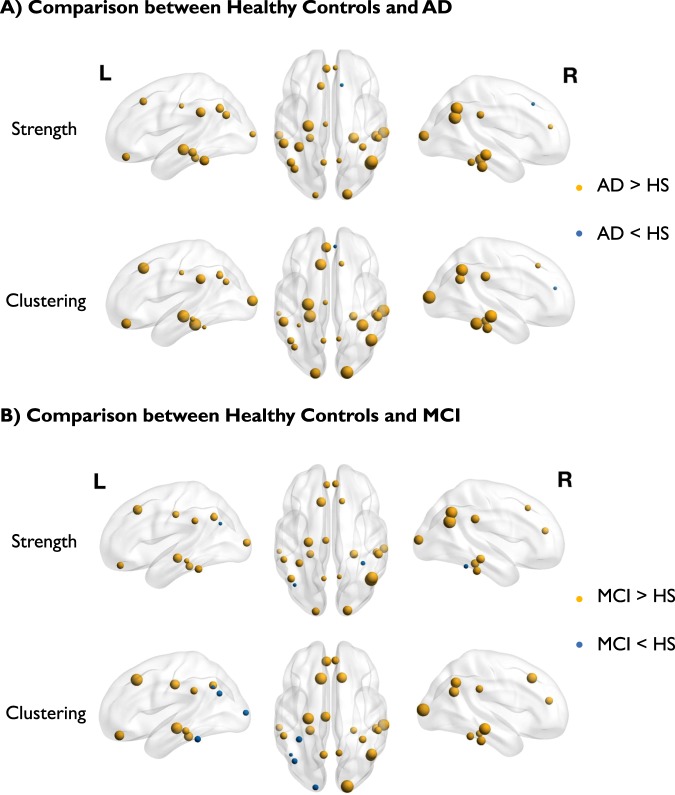
Graphical representation of strength and clustering analysis for [^18^ F]FDG PET data in AD (panel A) and MCI (panel B) subjects compared to healthy controls. Size of the spheres indicates the amplitude of the difference. Colour of the spheres indicates the direction of changes (yellow indicates increase in MCI/AD; blue indicates increase in healthy controls).

Figures 5 and 6 show the differences among the three groups (healthy controls, MCI and AD) found in the PET covariance analysis of the [^18^F]FDG images from the ADNI dataset. Comparisons between AD patients and healthy controls showed that both node strengths and clustering coefficients significantly increased in AD patients in mean (p-value < 0.01) but not in variance (p-value > 0.95). The ROIs with the highest increase in terms of strength were the hippocampus and the inferior parietal cortex (Fig. 6A). The ROIs with the highest increase in terms of clustering coefficient were the hippocampus and the mid-occipital cortex (Fig. 6A). No significant differences were found for the correlation distribution in terms of mean (p-value = 0.12) and variance (p-value = 0.99). Entropy was significantly increased for the AD patients (mean relative difference +41%; p-value < 0.01). The two PET adjacency matrices (AD vs controls) also differed in terms of principal component eigenvectors returned by the Krzanowski’s test (p-value < 0.01; Krzanowski’s *λ*: 7.47; Krzanowski’s *μ*: 8.09).

Comparisons between MCI subjects and healthy controls showed that node strength was the only metric to be significantly different between the two groups (mrd: +61%; p-value = 0.03), while all the other tests showed no significant differences. Similarly, the inferior parietal cortex showed the highest difference in strength between AD and controls, while the mid-occipital cortex showed the highest increase in terms of clustering coefficient (Fig. 6B).

Comparisons between MCI subjects and AD patients showed no significant differences for any test or metric, although there was a trend of increase in entropy in AD patients (+19%; p-value = 0.076) and the principal component eigenvalues were different at a trend level (Krzanowski’s test p-value = 0.064).

These results show a gradual change of the [^18^F]FDG brain PET covariance from healthy controls to the different stages of AD (Fig. 5A,B). Full results for all the cross-sectional comparisons are reported in the supplementary materials.

## Discussion

In this paper we proposed and tested a framework for the covariance analysis of brain PET data using graph-derived metrics and permutations statistics. The tool was applied to several PET datasets obtained from different PET tracers (i.e. [^18^F]FDG, [^18^F]FDOPA and [^11^C]SB207145), referring to different biological systems: glucose metabolism, dopamine synthesis and serotonin 5HT4 receptor availability. The results showed a good reproducibility of the method, thus supporting its general applicability within certain limits.

As to the method validation, in the resampling test no differences between the two groups were assumed as the groups were derived from the same population. The results showed that permutation tests perform better than the parametric tests in terms of FPR and threshold sensitivity. Of note, a higher FPR in the parametric test is unlikely to be a failure of the test itself, but a direct consequence of the data characteristics that do not meet the assumptions for its applicability. In fact, by using the Shapiro-Wilk normality test we found that the node strength and the clustering coefficient followed a normal distribution in less than 1% and 6% of the all the tested cases, respectively. Interestingly, the test performances were consistent across tracers (both parametric and permutation tests), supporting the applicability of PET covariance analysis to different kinetic parameters, number of subjects and brain ROIs. Surprisingly, our results did not show any association between PET population size and method performances, even with a sample of 10 subjects. However, the prediction of the statistical power for the interregional correlations calculated with such a small sample size is quite poor (*β* ~ 0.54). For datasets of this size, we strongly recommend the use of more robust methods^27,30^, while the current approach can be applied for a sample size ≥35, as shown by several examples in literature^10,25^. The test-retest analysis proved a very good reliability of this method in assessing the reproducibility of PET matrices and network metrics between test and retest conditions. The study related to the sensitivity of the graph analysis to the scanner type and image processing showed that the quality and resolution of the PET images returned by the scanners consistently affect the characteristics of the PET matrices. Both HRRT and partial-volume corrected data provided a more refined description of interregional correlation compared to the correspondent GE-Advance scans and partial-volume incorrected data, respectively.

The most interesting finding came from the clinical study related to the ADNI dataset. In fact, when comparing the PET covariance matrices between healthy controls and subjects at different stages of AD, a gradual change in the organization of brain glucose metabolism emerged. Our results show the highest changes in the hippocampus as well as in the inferior parietal cortex and mid-occipital cortex, a set of ROIs significantly linked to alterations of the glucose metabolism^53^. Although it is not the purpose of this paper to comment on the meaning of such findings in relation to AD pathology, it is important to note that our results confirm the previous evidence of differences in brain glucose metabolism and metabolic connectivity in AD. In 1987, Horwitz showed for the first time that Alzheimer patients had significantly fewer reliable [^18^F]FDG interregional brain correlations compared to healthy controls^11^. This result has been replicated over the years with different methods^18,30,54^ and even used for patient classification and prediction of conversion to AD^55^.

Furthermore, among the metrics and parameters extracted from the PET adjacency matrix, the entropy is the most novel and biologically informative statistics in the context of this clinical study and, in general, for the studies on ageing and neurodegeneration. In fact, the common denominator that underlies all modern theories of ageing is the change of the molecular structure turning into function decline^56^. This process occurs because the changed energy states of biomolecules make them inactive or malfunctioning. Every biological system tries to limit its energy dispersal through the continuous action of repair and replacement processes, aiming at maintaining the system homeostasis. In this perspective, entropy and its variations can be interpreted as fundamental measures of the system functioning and age respectively^57,58^. This relationship between entropy and biology has a particular value for neuroscience. For the central nervous system, an increased entropy is seen as one of the fundamental driving causes of neural and cognitive decline in the elderly^59^. In psychiatry, the value of entropy has been highlighted as a measure of defective development in psychosis^60,61^ and bipolar disorders^62^. Measuring brain entropy requires to know the energy state of all the molecules, cells, and tissues of the brain. This is impossible by definition as there are no techniques allowing to measure this quantity. Nevertheless, since entropy reflects the level of a system disorganization, the problem can be simplified by considering specific brain functional subsystems with metabolism being the most appropriate one. The key for entropy maintenance is an efficient production of energy. For the brain, as for any other key organ, energy supply is essential to explicate functions and to maintain homeostasis. The brain is also one of the most metabolically active organs in the body: although it represents only 2% of the body weight, it receives 15% of the cardiac output, 20% of total body oxygen consumption and 25% of total body glucose. For these reasons, the brain metabolic entropy could be an informative perspective of the true brain entropy. Our results on the entropy estimates obtained from [^18^F]FDG PET adjacency matrices showed an increase of this parameter in AD. If this metric can reflect both normal ageing and neurodegeneration of the brain, it might represent a potential biomarker to determine the normal and abnormal functioning of the central nervous system. One might also speculate that as entropy increases continuously over time, mirroring the age-changes that affect the molecular, cellular and brain functions^42,63^, it may predict the gradual transition from a healthy status to pathology, even before the onset of the disease. Given the heterogeneity of our MCI sample and the lack of information on their conversion to AD, we could not properly evaluate this hypothesis. Further studies in animal models and humans are needed.

### What is the added value of PET covariance analysis?

PET covariance statistics aims at assessing the functional organization of any biological function measured by a PET imaging radiotracer. This method is potentially very interesting as it provides information on brain organization at rest in pathophysiological conditions. The application of this method to investigate brain metabolic connectivity is only one applicative area of interest^19,21,29^. For example, the study of receptor functional organization might provide additional information into the understanding of biological mechanisms related to psychiatric disorders^64,65^.

PET covariance analysis is also interesting from a methodological point of view as it can provide a useful platform of integration with other neuroimaging modalities, particularly with MR-based techniques. Multimodal image covariance analysis might provide a new way to investigate the association between different processes measured by different neuroimaging techniques, accounting for the complexity of the entire brain rather than focusing on single regions. In this respect, independent component analysis (ICA) on intervoxel correlations would be a completely data-driven approach to identify functional networks without any a priori brain parcellation. The method has been successfully used to explore similarities and discrepancies between MRI functional connectivity and brain metabolism^17^. Further analysis on PET covariance statistics is likely to follow in this area with the increasing spread of multimodal brain acquisitions from PET-MR scanners^66,67^. Similarly, covariance PET analysis would allow the comparison of network properties across different tracers to explore interactions between different brain subsystems. This application could contribute to unveil fundamental mechanisms of brain function *in vivo* in humans, although more work on methodology development would be necessary.

### Limitations

This work presents a number of limitations, some of which are dependent on the choice of the PET tracers and experimental parameters used for the method validation. Others go beyond the particular characteristics of the data used in this study. First of all, this type of PET covariance analysis can only be done at the group level, as demonstrated in previous publications^10,18^. Moreover, it relies on the definition of a homogenous group of subjects and on the normalization of between-subject differences^10^, which ultimately reduces the method applicability. While the latter can be taken into account, removing inhomogeneities is more difficult as the dependency of the network metrics to these variables can become non-linear (see supplementary material). Moreover, the sensitivity of PET-derived network metrics might be different, highlighting the importance of matching the groups. For these reasons the ideal scenario would be to use groups made by the same subjects but at different experimental conditions (e.g. after a challenge/intervention or using different PET tracers). One alternative could be to use raw dynamic PET data with the advantage to define the PET covariance matrix at the subject level. This solution has already been used to compute temporal metabolic connectivity data^68,69^. However, raw regional kinetics carry additional information of tracer non-specific binding and delivery, hiding the tracer specific interaction with its targets. Further studies are needed to ascertain whether PET covariance analysis can be extended to an individual basis.

Another important limitation is the sensitivity of the method to the image resolution. This variable is controlled by several experimental parameters (including scanner type and reconstruction method) as well as by the image pre-processing pipeline (including segmentation, denoising and smoothing). All these elements intrinsically bias the interregional correlations especially in those regions that share a boundary, modifying the topological characteristics of the corresponding PET adjacency matrix. Even if we tested the method in brain PET data sets using different brain segmentations, it remains unclear how region size and partial volume might affect the PET covariance cross-sectional statistics. Extending the method at the voxel level could be a possibility to partially mitigate the problem, although permutation testing could become computationally too heavy and thus not applicable.

If different settings are used within the same group of subjects, it is not possible to covary for them as it is normally done in a more conventional statistical PET analysis. Firstly, it is difficult to model the effects that a variable might have on the interregional correlations. Secondly, the statistical comparison is based on permutation testing, which requires to account for different experimental variables during the randomization process. Therefore, it becomes important to remove any ancillary covariates from the PET data before the analysis of the PET covariance matrix.

The definition of the PET adjacency matrix is also another source of variability in neuroimaging covariance analysis: simple correlation, partial correlation, sparse inverse covariance estimation, multivariate distant correlation can all be applied to generate these matrices^10,30,70,71^. Voxel-based approaches rather than region-based one are also available^16,18^. Since there is no agreement on the method to use, one should use the most appropriate depending on the research question. Similarly, there are many different ways to statistically compare PET covariance matrices or PET-derived networks. Despite the multitude of solutions, our results clearly show that these objects do not fit the assumptions for the application of parametric tests. Hence the use of permutations for deriving the null distribution becomes essential. Of note, permutation testing does not avoid the problem of multiple comparisons. Several methods for multiple comparison correction are available for permutation testing depending on the type of neuroimaging data and on the research question (i.e. exploratory study vs. hypothesis-driven study)^72,73^. Further work is needed to integrate multiple comparison correction with PET covariance analysis.

## Conclusions

Covariance statistics and network-based methods can be applied to brain PET studies to investigate the topological characteristics of the tracer kinetics and its related targets. This information can be used to understand how biological functions are organised across brain regions in healthy and pathological conditions. The proposed method is complementary to the more standard PET analysis, where cross-sectional mean differences of tracer kinetics are generally the only outcomes. Results are highly dependent on experimental design and variables, including group inhomogeneity and image resolution, and further methodological work is required to validate the use of more complex network metrics in the context of PET covariance analysis and to understand their biological interpretability. Nonetheless this approach has a great potential for the analysis of multimodal/multitracer neuroimaging studies.

## Supplementary information

Supplementary Material

## Data Availability

All the analyses were run using NetPET, an in-house developed software package running on Matlab (Mathworks®). Download is available at: http://www.nitrc.org/projects/netpet_2018/. The code used to calculate the network measures utilised Matlab BCT original implementation^33^. The PET data that support the findings of this study are available from multiple resources which includes the ADNI database (adni.loni.usc.edu), the CIMBI database (https://nru.dk/index.php/research-menu/115-the-cimbi-database-and-biobank) and from the internal institutional PET repository. Some restrictions apply to the availability of these data, which were used under license for the current study, and so are not publicly available. Data are available upon reasonable request.
