# Supplementary material for: Covariance statistics and network analysis of brain PET imaging studies
Source: Sci Rep. 2019 Feb 21;9:2496. doi: 10.1038/s41598-019-39005-8 (PMC6385265; doi:10.1038/s41598-019-39005-8)
Supplement: Supplementary file 1 — Supplementary Material [file 41598_2019_39005_MOESM1_ESM.docx]

**Covariance statistics and network analysis of brain PET imaging studies**

Mattia Veronese^1^, Lucia Moro ^1,2^, Marco Arcolin^1,2^, Ottavia Dipasquale^1^, Gaia Rizzo^3^, Paul Expert^4,5^, Wasim Khan^1,6^, Patrick M. Fisher^7^, Claus Svarer^7^, Alessandra Bertoldo^2^, Oliver Howes^8^, Federico E. Turkheimer^1^

Affiliations:

^1^Department of Neuroimaging, IoPPN, King’s College London, London, UK

^2^Department of Information Engineering, University of Padova, Padova, Italy

^3^Invicro UK, London, UK

^4^Department of Mathematics, Imperial College London, London

^5^EPSRC Centre for Mathematics of Precision Healthcare, Imperial College London

^6^Florey Institute of Neuroscience and Mental Health, Melbourne Brain Centre, Melbourne, Australia

*^7^Neurobiology Research Unit, Copenhagen University Hospital Rigshospitalet, Copenhagen, DK*

*^8^Department of Psychosis studies, IoPPN, King’s College London, London, UK*

**SUPPLEMENTARY MATERIAL**

**Entropy**

In addition to the node strength and clustering coefficient, we also computed the functional Entropy ([Yao, Lu et al. 2013](#_ENREF_10)) of the PET adjacency matrix. Entropy is a global measure used for estimating the uncertainty about the state of a system. This measure has already been applied to biological systems ([Hayflick 2007](#_ENREF_4)) including the brain ([Carhart-Harris, Leech et al. 2014](#_ENREF_1)), with low entropy corresponding to a high level of predictability and high entropy corresponding to a high level of randomness. Functional Entropy ($H$) was defined as

| $H=-\sum_{i=1}^{N} p_{i}{log}_{2}p_{i}$ | (1) |
| --- | --- |

where $p_{i}$ is the probability of the $i$-th sample of the interregional correlation distribution derived from the PET adjacency matrix and $N$ its number of bins. This approach has already been used for the analysis of resting-state functional MRI data ([Yao, Lu et al. 2013](#_ENREF_10)). Given the discrete nature of covariance matrix distribution, $N$ was defined based on the Scott and Terrell criteria ([Terrell and Scott 1985](#_ENREF_9)).

Entropy differences were assessed by

| $t_{H}= \frac{H_{1}- H_{2}}{\sqrt{({Hvar}_{1}-{Hvar}_{2})}}$ | (2) |
| --- | --- |

where $H_{1}$ and $H_{2}$ represents the entropy estimates of the two groups respectively, and ${Hvar}_{1}$ and ${Hvar}_{2}$ their variances as computed by Hutcheson formula ([Hutcheson 1970](#_ENREF_5)). The entropy statistic $t_{H}$ was then compared against the distribution of entropy measures $\hat{t_{H}}$ generated by 10,000 random permutations of the dataset in two groups of similar dimensions as the original ones. The p-value was approximated by the fraction of permutations with correspondent entropy statistics greater than the original one in absolute value (i.e. $\left| \hat{t_{H}} \right|\geq\left| t_{H} \right|$). Five percent was used as statistical threshold.

**TEST ON PRINCIPAL COMPONENTS**

A commonly used technique for exploring a multivariate dataset is principal component analysis. The Krzanowski’s test on the principal components of the PET adjacency matrixes was hence applied to investigate the equality of their eigenvectors and eigenvalues ([Krzanowski 1993](#_ENREF_7)). The test is based on a permutation approach described as follows. Given two populations of $n1$ and $n2$ individuals of which the same $t$variables are measured (in our case $t$ corresponds to the number of ROIs), let $R1$ and $R2$ be the correspondent correlation matrices. For $R1$ and $R2$ their eigenvalues and eigenvectors can be computed. Let the eigenvalues of $R1$ be stored in ascending order in the matrix $D1$ ($D2$ for $R2$) and the corresponding eigenvectors in successive columns of $M$ ($L$ for $R2$). As for $L_{(k)}$ and $M_{(k)}$, the first $k$ components of $L$ and $M$ are indicated, with $k$ being the largest integer number smaller than $t/2$. Krzanowski (1979) has shown that the inverse cosine of the square roots of the eigenvalues of

| ${L_{(k)}}^{T}M_{(k)}{M_{(k)}}^{T}L_{(k)}$ | (3) |
| --- | --- |

gives the size of the angles between the subspace defined by $L_{(k)}$ and $M_{(k)}$. The information about the amplitude of these angles can be combined into a single value, $\lambda$ as the sum of their square cosines*.*

To test the null hypothesis that the populations from which the $R1$ and $R2$ have the same principal components, the subjects for *n*1 and *n*2 are randomly reassigned to two groups of the same dimension as the original ones. For each $i$-th permutation the two matrices $L_{(k)}^{i}$ and $M_{(k)}^{i}$ can be generated and a null distribution defined ($\hat{\lambda}$). By comparing the original $\lambda$ with $\hat{\lambda}$the test determines whether the principal components of the two matrices differ in eigenvectors. If they do not differ, Krzanowski’s test continue testing for the eigenvalues, by comparing the parameter

| $\mu=trace(\left\vert D1-D2 \right\vert)$ | (4) |
| --- | --- |

with the corresponds null distribution $\hat{\mu}$ obtained from the data permutation in a similar way of $\hat{\lambda}$. If both tests return non-statistically significant differences, the null hypothesis (two matrices are equivalent) is retained.

**REFERENCES:**

Carhart-Harris, R. L., R. Leech, P. J. Hellyer, M. Shanahan, A. Feilding, E. Tagliazucchi, D. R. Chialvo and D. Nutt (2014). "The entropic brain: a theory of conscious states informed by neuroimaging research with psychedelic drugs." Frontiers in human neuroscience **8**.

Egerton, A., A. Demjaha, P. McGuire, M. A. Mehta and O. D. Howes (2010). "The test–retest reliability of 18F-DOPA PET in assessing striatal and extrastriatal presynaptic dopaminergic function." Neuroimage **50**(2): 524-531.

Hammers, A., R. Allom, M. J. Koepp, S. L. Free, R. Myers, L. Lemieux, T. N. Mitchell, D. J. Brooks and J. S. Duncan (2003). "Three‐dimensional maximum probability atlas of the human brain, with particular reference to the temporal lobe." Human brain mapping **19**(4): 224-247.

Hayflick, L. (2007). "Entropy explains aging, genetic determinism explains longevity, and undefined terminology explains misunderstanding both." PLoS genet **3**(12): e220.

Hutcheson, K. (1970). "A test for comparing diversities based on the Shannon formula." Journal of theoretical Biology **29**(1): 151-154.

Knudsen, G. M., P. S. Jensen, D. Erritzoe, W. F. Baaré, A. Ettrup, P. M. Fisher, N. Gillings, H. D. Hansen, L. K. Hansen and S. G. Hasselbalch (2016). "The center for integrated molecular brain imaging (Cimbi) database." Neuroimage **124**: 1213-1219.

Krzanowski, W. (1993). "Permutational tests for correlation matrices." Statistics and Computing **3**(1): 37-44.

Martinez, D., M. Slifstein, A. Broft, O. Mawlawi, D.-R. Hwang, Y. Huang, T. Cooper, L. Kegeles, E. Zarahn and A. Abi-Dargham (2003). "Imaging human mesolimbic dopamine transmission with positron emission tomography. Part II: amphetamine-induced dopamine release in the functional subdivisions of the striatum." Journal of Cerebral Blood Flow & Metabolism **23**(3): 285-300.

Terrell, G. R. and D. W. Scott (1985). "Oversmoothed nonparametric density estimates." Journal of the American Statistical Association **80**(389): 209-214.

Yao, Y., W. Lu, B. Xu, C. Li, C. Lin, D. Waxman and J. Feng (2013). "The increase of the functional entropy of the human brain with age." Scientific reports **3**: 2853.

**Test-retest analysis**

1. [^18^F]FDOPA dataset (Group 1: baseline; Group 2: rescan)

*Covariance metrics analysis*

|  | *MEAN* | | *VARIANCE* | | *p-value* | |
| --- | --- | --- | --- | --- | --- | --- |
|  | *Group 1* | *Group 2* | *Group 1* | *Group 2* | *MEAN* | *VARIANCE* |
| **Correlation** | -0.002 | -0.004 | 0.272 | 0.381 | 0.877 | 0.938 |
| **Strength** | 4.653 | 8.334 | 8.472 | 47.96 | 0.172 | 0.986 |
| **Clustering** | 0.362 | 0.542 | 0.075 | 0.169 | 0.147 | 0.992 |
|  | *Statistics* | | *p-value* | |  |  |
|  | *Lambda* | *Mu* | *Eigenvector* | *Eigenvalue* |  |  |
| **KRZONOWSKY** | 10.672 | 8.829 | 0.110 | 0.430 |  |  |
|  | *Group 1* | *Group 2* | *Statistics* | *p-value* |  |  |
| **Entropy** | 3.992 | 4.330 | -12.644 | 0.134 |  |  |

*Correlation matrixes*


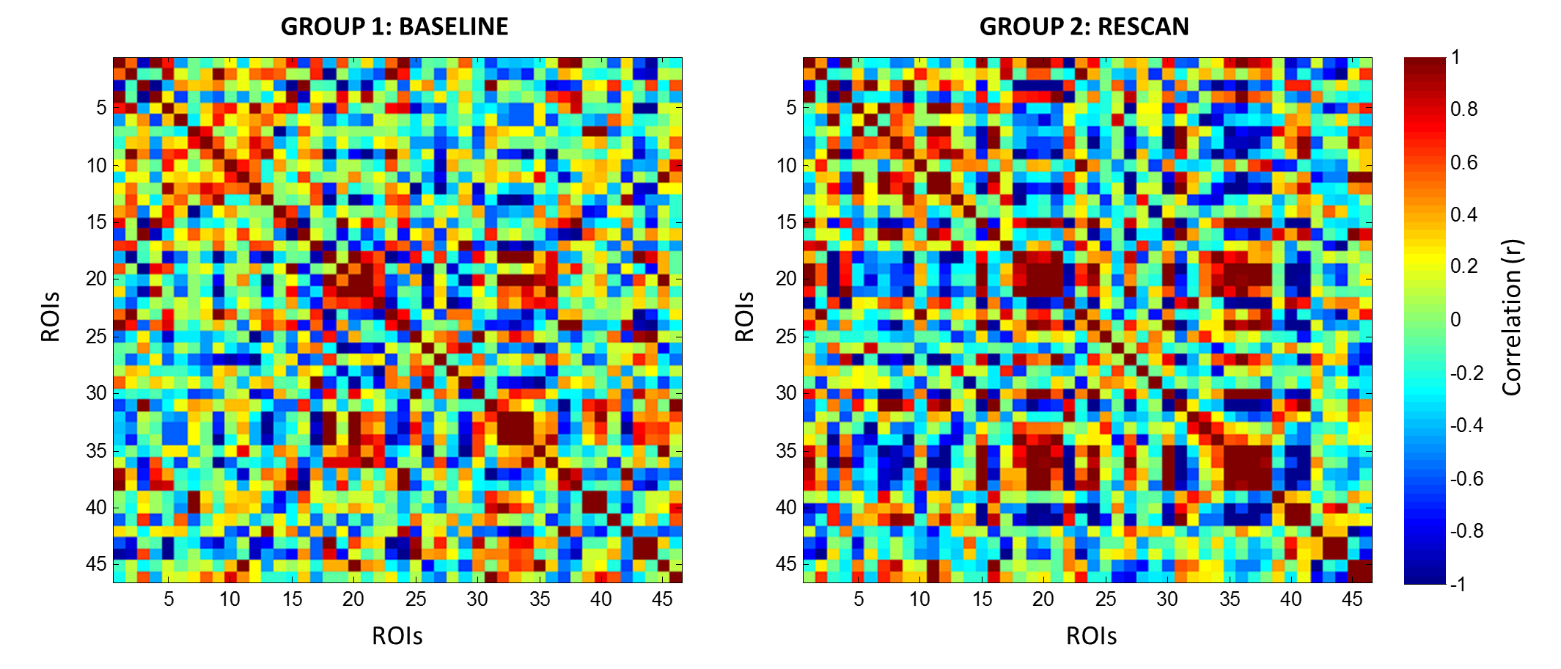


*ROIs (in order of representations): Whole_striatum, Whole_sensorimotor_striatum_Subdivision, Whole_limbic_striatum_Subdivision, Whole_Associative_Subdivision (from Di Martinez Atlas (*[*Martinez, Slifstein et al. 2003*](#_ENREF_8)*)), HIPPOCAMPUS, AMYGDALA, ANT_TL_MED, ANT_TL_INF_LAT, G_PARAH_AMB, , G_SUP_TEMP_CENT, G_TEM_MIDIN, G_OCCTEM_LA, BRAINSTEM, INSULA, OL_REST_LAT, G_CING_ANT_SUP, G_CING_POST, FL_MID_FR_G, POSTERIORTL, PL_REST, CAUDATENUCL, NUCLACCUMB, PUTAMEN, THALAMUS, PALLIDUM, CORPUM_CALLOSUM, FRONTALHORN, TEMPORAHORN, THIRDVENTRICL, FL_PRECEN_G, FL_STRAI_G, FL_OFC_AOG, FL_INF_FR_G, FL_SUP_FR_G, PL_POSTCE_G, PL_SUP_PA_G, OL_LING_G, OL_CUNEUS, FL_OFC_MOG, FL_OFC_LOG, FL_OFC_POG, S_NIGRA, SUBGEN_ANTCING, SUBCALL_AREA, PRESUBGEN_ANTCING, G_SUP_TEMP_ANT (from Hammerstmith Atlas (*[*Hammers, Allom et al. 2003*](#_ENREF_3)*)). Full details of ROI description are reported in (*[*Egerton, Demjaha et al. 2010*](#_ENREF_2)*).*

1. [^11^C]SB207145 dataset (Group 1: baseline; Group 2: rescan)

*Covariance metrics analysis*

|  | *MEAN* | | *VARIANCE* | | *p-value* | |
| --- | --- | --- | --- | --- | --- | --- |
|  | *Group 1* | *Group 2* | *Group 1* | *Group 2* | *MEAN* | *VARIANCE* |
| **Correlation** | 0.075 | 0.077 | 0.311 | 0.416 | 0.930 | 0.914 |
| **Strength** | 20.80 | 28.30 | 149.85 | 162.80 | 0.095 | 0.911 |
| **Clustering** | 0.519 | 0.614 | 0.016 | 0.020 | 0.153 | 0.619 |
|  | *Statistics* | | *p-value* | |  |  |
|  | *Lambda* | *Mu* | *Eigenvector* | *Eigenvalue* |  |  |
| **KRZONOWSKY** | 26.389 | 14.218 | 0.910 | 0.103 |  |  |
|  | *Group 1* | *Group 2* | *Statistics* | *p-value* |  |  |
| **Entropy** | 4.299 | 4.743 | -20.080 | 0.084 |  |  |

*Correlation matrixes*


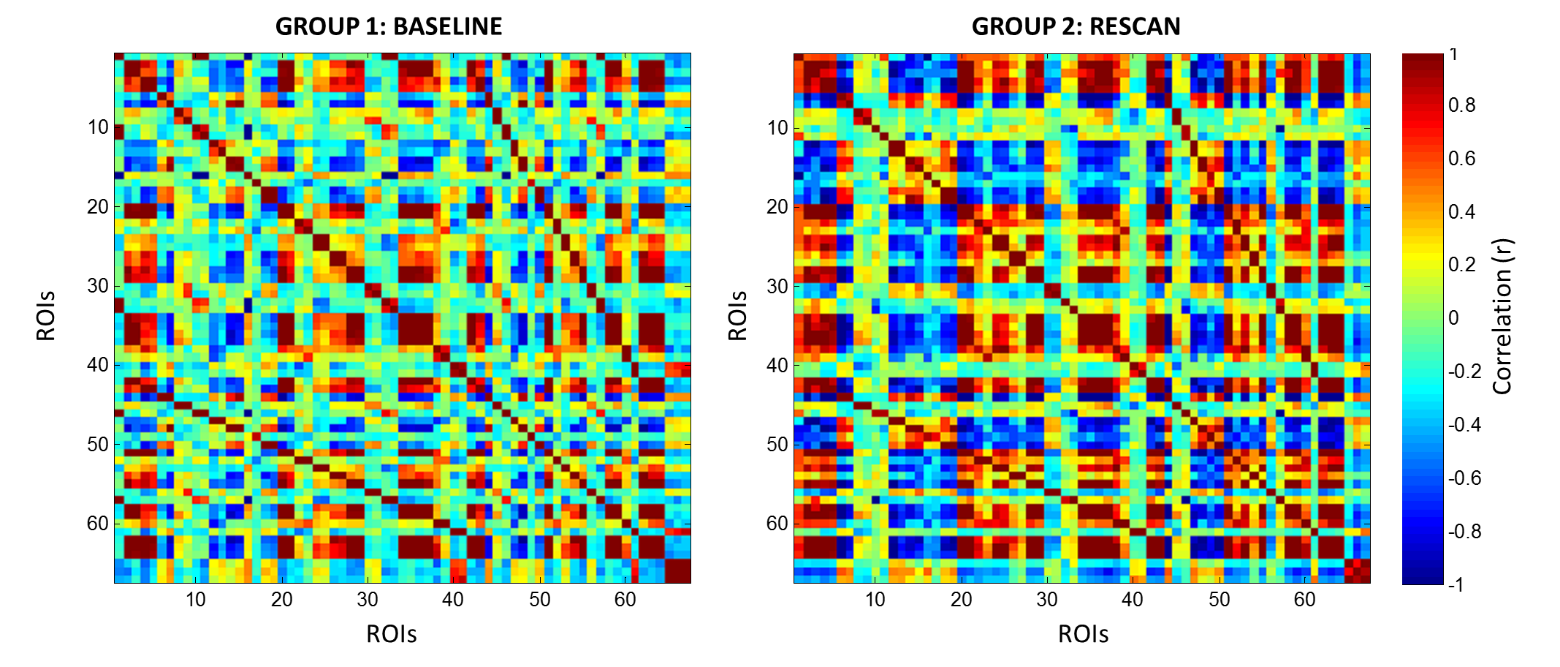


*The ROI list includes Dorso Lateral Pre Frontal Cortex, Ventro Lateral Pre Frontal Cortex, Amygdala, Anterior, Cingulate, Caudatum, Entorhinal Cortex, Hippocampus, Hypothalamus, Insula, Medial Inferior Gyrus, Medial Inferior Temporal Gyrus, Occipital, Orbital Frontal Cortex, Paracentral, Posterior Cingulate, Putamen, Sensory Motor Cortex, Superior Frontal Gyrus, Superior Temporal Gyrus, Thalamus. Each ROI is reported Left and Right separately and combined, for 67 ROIs overall. Full details of ROIs is reported in (*[*Knudsen, Jensen et al. 2016*](#_ENREF_6)*).*

**Sensitivity to scanner type and partial volume**

1. [^11^C]SB207145 dataset (Group 1: HRRT; Group 2: GE-Advance)

*Covariance metrics analysis*

|  | *MEAN* | | *VARIANCE* | | *p-value* | |
| --- | --- | --- | --- | --- | --- | --- |
|  | *Group 1* | *Group 2* | *Group 1* | *Group 2* | *MEAN* | *VARIANCE* |
| **Correlation** | 0.048 | 0.102 | 0.162 | 0.395 | 0.013 | 1.000 |
| **Strength** | 13.024 | 25.891 | 47.57 | 48.67 | <0.001 | 1.000 |
| **Clustering** | 0.366 | 0.595 | 0.016 | 0.016 | <0.001 | 0.449 |
|  | *Statistics* | | *p-value* | |  |  |
|  | *Lambda* | *Mu* | *Eigenvector* | *Eigenvalue* |  |  |
| **KRZONOWSKY** | 24.755 | 24.381 | <0.001 | NaN |  |  |
|  | *Group 1* | *Group 2* | *Statistics* | *p-value* |  |  |
| **Entropy** | 3.558 | 4.609 | -37.940 | <0.001 |  |  |

*Correlation matrixes*


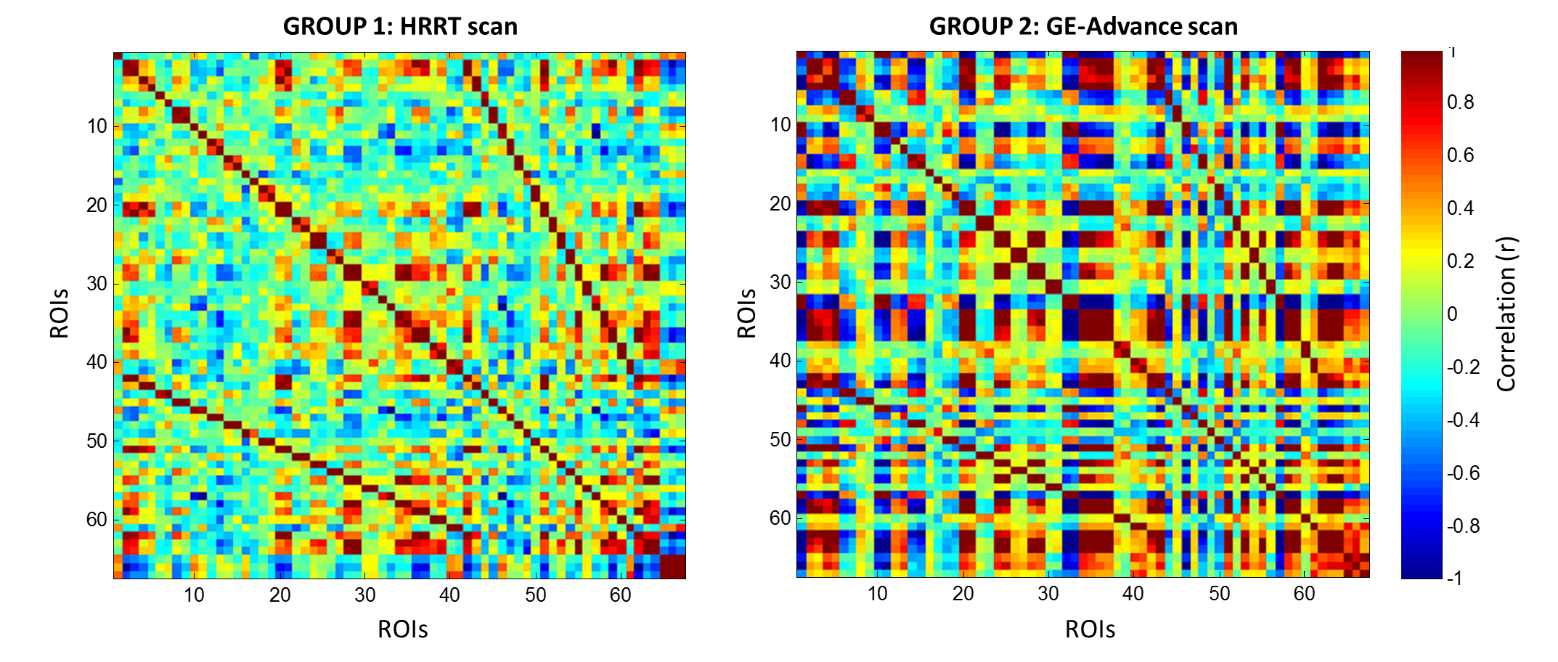


*The ROI list includes Dorso Lateral Pre Frontal Cortex, Ventro Lateral Pre Frontal Cortex, Amygdala, Anterior, Cingulate, Caudatum, Entorhinal Cortex, Hippocampus, Hypothalamus, Insula, Medial Inferior Gyrus, Medial Inferior Temporal Gyrus, Occipital, Orbital Frontal Cortex, Paracentral, Posterior Cingulate, Putamen, Sensory Motor Cortex, Superior Frontal Gyrus, Superior Temporal Gyrus, Thalamus. Each ROI is reported Left and Right separately and combined, for 67 ROIs overall. Full details of ROIs is reported in (*[*Knudsen, Jensen et al. 2016*](#_ENREF_6)*).*

1. [^11^C]SB207145 dataset (Group 1: PVC; Group 2: Not PVC)

*Covariance metrics analysis*

|  | *MEAN* | | *VARIANCE* | | *p-value* | |
| --- | --- | --- | --- | --- | --- | --- |
|  | *Group 1* | *Group 2* | *Group 1* | *Group 2* | *MEAN* | *VARIANCE* |
| **Correlation** | 0.119 | 0.102 | 0.241 | 0.377 | 0.419 | 1.000 |
| **Strength** | 19.62 | 23.95 | 47.40 | 47.52 | 0.031 | 1.000 |
| **Clustering** | 0.398 | 0.536 | 0.003 | 0.027 | <0.001 | 1.000 |
|  | *Statistics* | | *p-value* | |  |  |
|  | *Lambda* | *Mu* | *Eigenvector* | *Eigenvalue* |  |  |
| **KRZONOWSKY** | 26.141 | 16.082 | 0.472 | <0.001 |  |  |
|  | *Group 1* | *Group 2* | *Statistics* | *p-value* |  |  |
| **Entropy** | 4.108 | 4.424 | -11.541 | 0.010 |  |  |

*Correlation matrixes*


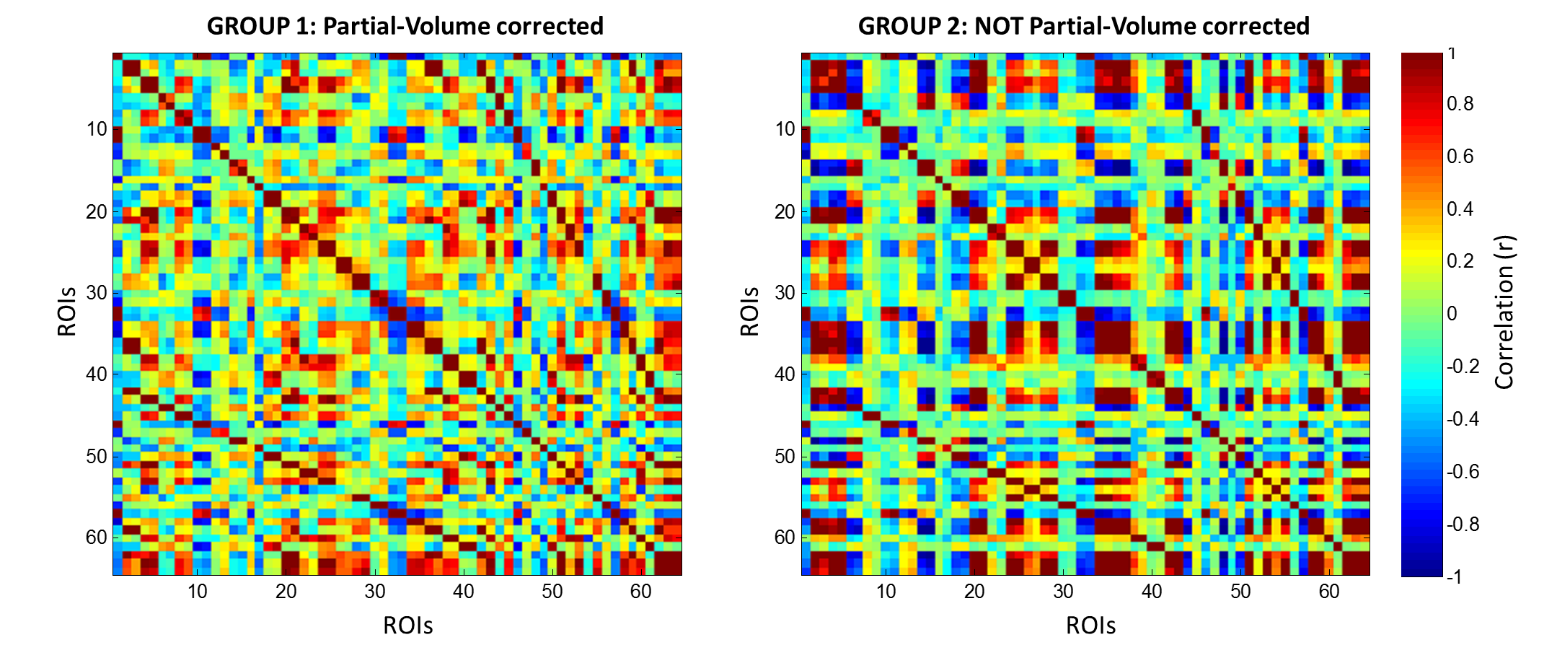


*The ROI list includes Dorso Lateral Pre Frontal Cortex, Ventro Lateral Pre Frontal Cortex, Amygdala, Anterior, Cingulate, Caudatum, Entorhinal Cortex, Hippocampus, Hypothalamus, Insula, Medial Inferior Gyrus, Medial Inferior Temporal Gyrus, Occipital, Orbital Frontal Cortex, Paracentral, Posterior Cingulate, Putamen, Sensory Motor Cortex, Superior Frontal Gyrus, Superior Temporal Gyrus, Thalamus. Each ROI is reported Left and Right separately and combined, for 67 ROIs overall. Full details of ROIs is reported in (*[*Knudsen, Jensen et al. 2016*](#_ENREF_6)*).*

**Application to Alzheimer’s Disease dataset**

1. [^18^F]FDG dataset (Group 1: healthy controls; Group 2: AD patients)

*Covariance metrics analysis*

|  | *MEAN* | | *VARIANCE* | | *p-value* | |
| --- | --- | --- | --- | --- | --- | --- |
|  | *Group 1* | *Group 2* | *Group 1* | *Group 2* | *MEAN* | *VARIANCE* |
| **Correlation** | -0.043 | -0.038 | 0.074 | 0.177 | 0.118 | 0.993 |
| **Strength** | 3.244 | 5.957 | 1.241 | 3.682 | <0.001 | 0.985 |
| **Clustering** | 0.187 | 0.317 | 0.003 | 0.009 | 0.001 | 0.950 |
|  | *Statistics* | | *p-value* | |  |  |
|  | *Lambda* | *Mu* | *Eigenvector* | *Eigenvalue* |  |  |
| **KRZONOWSKY** | 7.468 | 8.089 | <0.001 | NaN |  |  |
|  | *Group 1* | *Group 2* | *Statistics* | *p-value* |  |  |
| **Entropy** | 1.806 | 2.743 | -10.349 | <0.001 |  |  |

1. [^18^F]FDG dataset (Group 1: healthy controls; Group 2: MCI)

*Covariance metrics analysis*

|  | *MEAN* | | *VARIANCE* | | *p-value* | |
| --- | --- | --- | --- | --- | --- | --- |
|  | *Group 1* | *Group 2* | *Group 1* | *Group 2* | *MEAN* | *VARIANCE* |
| **Correlation** | -0.043 | -0.043 | 0.074 | 0.111 | 0.800 | 0.964 |
| **Strength** | 3.244 | 5.244 | 1.241 | 2.910 | 0.027 | 0.915 |
| **Clustering** | 0.187 | 0.246 | 0.003 | 0.003 | 0.320 | 0.740 |
|  | *Statistics* | | *p-value* | |  |  |
|  | *Lambda* | *Mu* | *Eigenvector* | *Eigenvalue* |  |  |
| **KRZONOWSKY** | 8.425 | 3.783 | 0.509 | 0.261 |  |  |
|  | *Group 1* | *Group 2* | *Statistics* | *p-value* |  |  |
| **Entropy** | 1.806 | 2.263 | -5.250 | 0.250 |  |  |

1. [^18^F]FDG dataset (Group 1: MCI; Group 2: AD patients)

*Covariance metrics analysis*

|  | *MEAN* | | *VARIANCE* | | *p-value* | |
| --- | --- | --- | --- | --- | --- | --- |
|  | *Group 1* | *Group 2* | *Group 1* | *Group 2* | *MEAN* | *VARIANCE* |
| **Correlation** | -0.043 | -0.038 | 0.111 | 0.177 | 0.051 | 0.983 |
| **Strength** | 5.244 | 5.957 | 2.910 | 3.682 | 0.354 | 0.547 |
| **Clustering** | 0.246 | 0.317 | 0.003 | 0.009 | 0.101 | 0.935 |
|  | *Statistics* | | *p-value* | |  |  |
|  | *Lambda* | *Mu* | *Eigenvector* | *Eigenvalue* |  |  |
| **KRZONOWSKY** | 8.426 | 4.456 | 0.197 | 0.064 |  |  |
|  | *Group 1* | *Group 2* | *Statistics* | *p-value* |  |  |
| **Entropy** | 2.263 | 2.743 | -5.727 | 0.076 |  |  |

**Sensitivity of PET-derived graph metrics to group age**


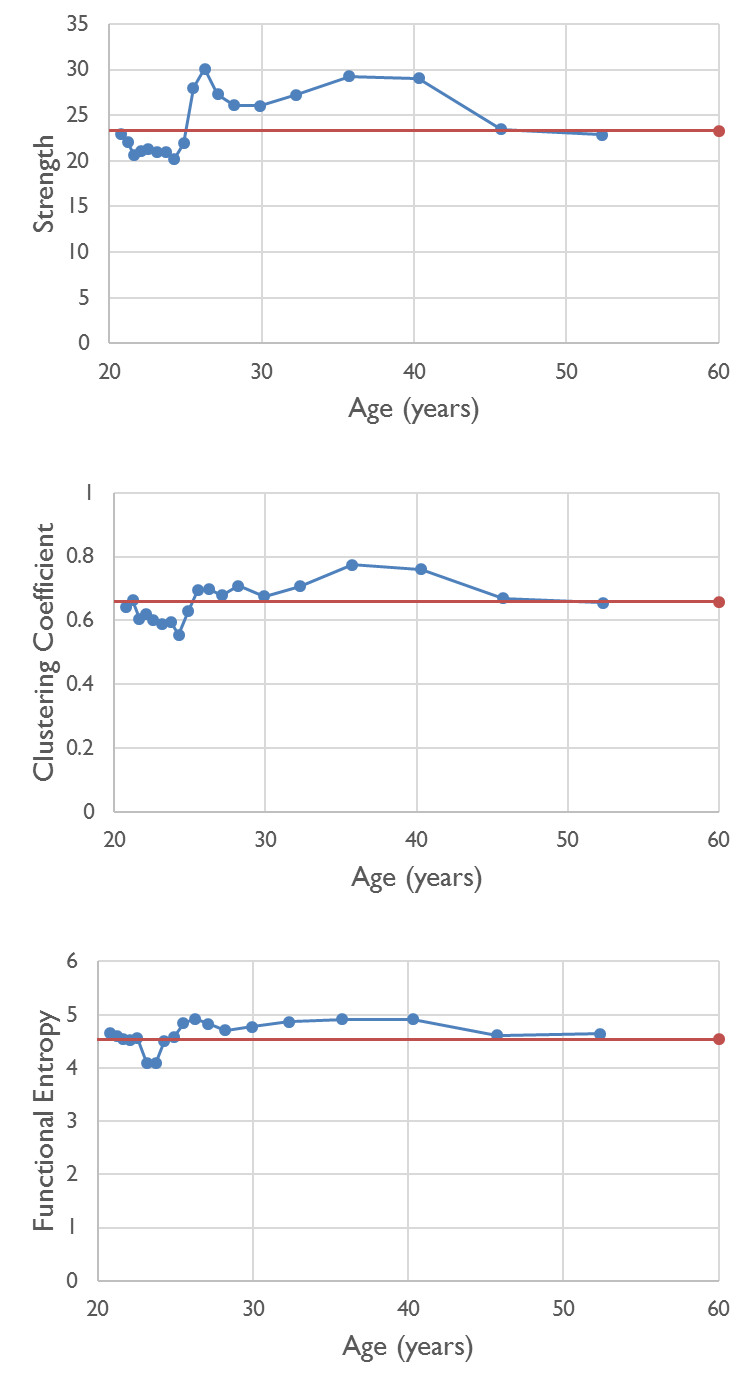
The graphs show the dependency of average node strength, average clustering coefficient and functional entropy on the group age (mean across subjects). The results were obtained by applying PET covariance statistics in different subgroups obtained from the [11C]SB217045 dataset with a similar procedure to that used for *moving average*. First, individuals were sorted from the youngest to the oldest. From this sorted sample, subgroups were defined by selecting sets of 20 consecutive subjects with increasing age. Overlap between subgroups was set to 75%. Blue lines show the metric values as function of the group age. Red lines indicate the metric values obtained from the whole sample analysis. It is important to note that, despite the overlap between adjacent subgroups, the variance of the samples is not constant across the samples but actually increases with the mean age of the subgroups.


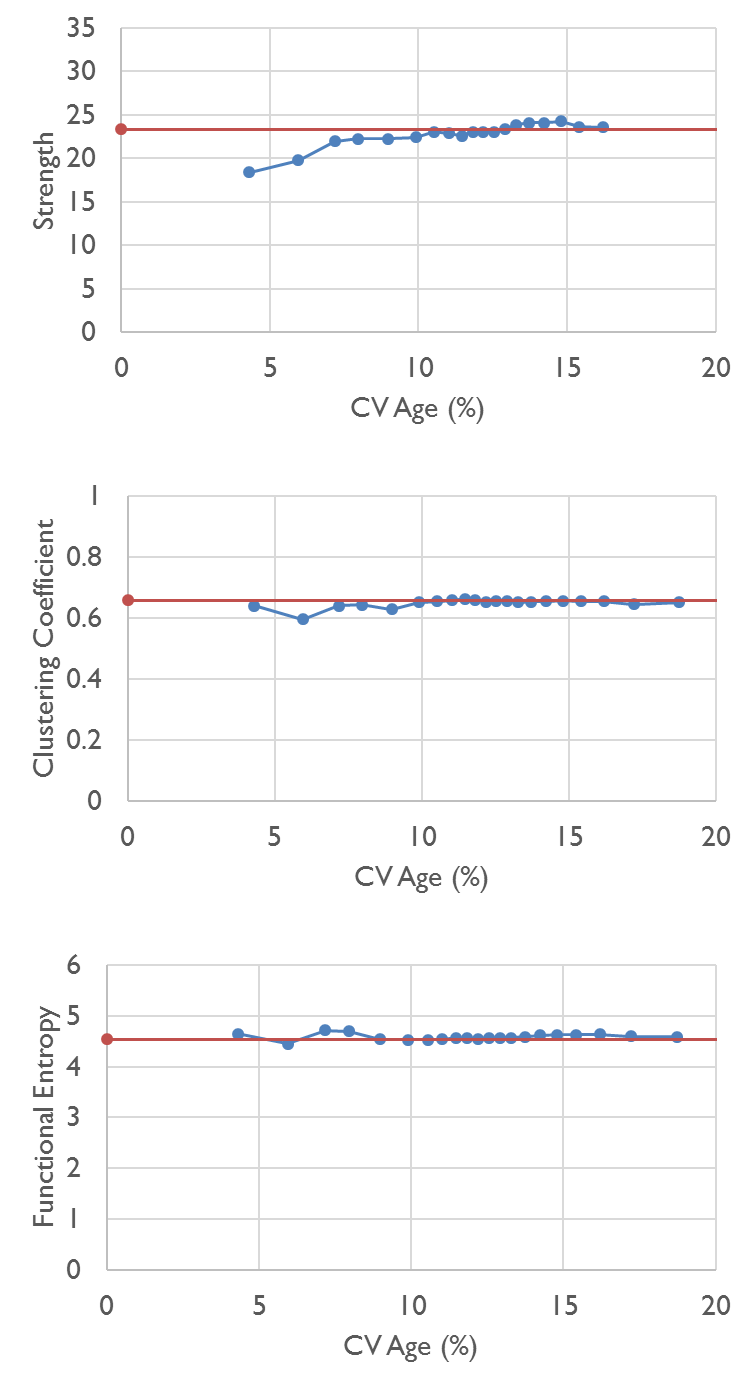
**Sensitivity of PET-derived graph metrics to group age variance**

The graphs show the dependency of average node strength, average clustering coefficient and functional entropy on the group age variance. The results were obtained by applying PET covariance statistics in different subgroups obtained from the [11C]SB217045 dataset. For each subgroup individuals were randomly chosen from the whole sample to maintain the subgroup age average constant to the whole population value (~23.5±0.5 years) but with increasing age variances. Age variances were selected from ~5% to ~20%. For each subgroup the number of individuals was maintained constant to 20 subjects as for the previous analysis on group age. The procedure was repeated 1,000 times. Blue lines show the metric values (average across 1,000 attempts) as function of the group age variance. Red lines indicate the metric values obtained from the whole sample analysis.
